# Perceived Links Between Playing Surfaces and Injury: a Worldwide Study of Elite Association Football Players

**DOI:** 10.1186/s40798-018-0155-y

**Published:** 2018-08-20

**Authors:** Aimée C. Mears, Paul Osei-Owusu, Andy R. Harland, Alun Owen, Jonathan R. Roberts

**Affiliations:** 1 Sports Technology Institute, Wolfson School of Mechanical, Electrical and Manufacturing Engineering, Loughborough University, Loughborough, UK; 2 Faculty of Engineering, Environment and Computing, Coventry University, Coventry, UK

**Keywords:** Soccer, Football playing surfaces, Injury, Perception, Surface properties, Questionnaire

## Abstract

**Background:**

Injuries in association football (soccer) are debilitating for players and can also be detrimental to the success of a team or club. The type or condition of a playing surface has been empirically linked to injuries, yet results are inconclusive. The overall purpose of this study was to analyse elite football players’ perceived links between playing surfaces and injury from a worldwide cohort of players. The results of this study can help to inform areas for future playing surface research aimed at trying to alleviate user concerns and meet user (i.e. the player) needs.

**Methods:**

Quantitative data were collected from 1129 players across the globe to address the aim of this study.

**Results:**

Ninety-one percent of players believed the type or condition of a surface could increase injury risk. Abrasive injuries, along with soreness and pain, were perceived to be greater on artificial turf. Surface type, surface properties and age were all potential risk factors identified by the players and linked to the playing surfaces.

**Conclusions:**

The results identified three areas where future research should be focussed to help develop surfaces that alleviate user concerns and meet user (i.e. player) needs: (i) current reporting of soreness, pain or fatigue as injuries, (ii) contribution of surface properties to injury; and (iii) surface experience of players from different countries differentiates their views of injury risk.

## Key Points

Analysis of the perceived links between playing surfaces and injury in a worldwide cohort of elite players revealed three areas where future research should be focussed to ensure playing surfaces consider the players’ concerns and meet the needs of the players:

•Current reporting of soreness, pain or fatigue as injuries

•Surface properties forming focus for future injury comparison studies rather than discrete surface types

•Targeting countries with similar player experience of surfaces

## Background

Injuries in association football (soccer) are debilitating for the player affected but also have a wider impact on team performances and morale and place substantial financial burdens on clubs [1]. Injuries in football have been empirically linked to the type or condition of a playing surface based on questionnaires of player perceptions [2, 3] and analysis of injury or medical reports [4, 5]. Notably, players have expressed negative attitudes towards the use of artificial turf (AT) (defined in this study as any synthetic grass football surface) for training and matches due to the perceived risk of injury [3]. Yet critical reviews of injury report studies have not found conclusive evidence that one particular surface increases the risk of injury over another even when comparing the severity of an injury (quantified as missed training or match days) or types of injury [6–9]. Therefore, there is still a need to assess players’ perception of the suitability of playing surfaces on important themes such as injury to help develop surface or future surface research that can alleviate user concerns and meet user needs.

The lack of conclusive evidence from injury reporting studies are due in part to different definitions of injury, study designs, data collection methods and length of injury observation [10–12]. Despite studies reporting no difference in the incidence of injury between AT and natural turf (NT), the avoidance of using AT in elite football, particularly the male game in Southern Europe, is often attributed to players’ continued negative perceptions of AT [8]. Poulos et al. [2] reported approximately 90% agreement by elite North American players (*n* = 99) that the type and quality of a playing surface could impact the risk of sustaining an injury and that the perceived risk was higher on AT. The players also self-reported longer recovery times after games and training on AT which was attributed to their increased feelings of joint and muscle soreness. Semi-professional Spanish footballers (*n* = 627) were largely dissatisfied with skin abrasions in sliding tackles (39.6%), playing at high temperatures (15.8%) and risk of sustaining an injury (10.6%) on AT compared to natural turf, yet opinions varied depending on age and surface experience [3]. Conversely, Zanetti [13] reported favourable attitudes towards AT over NT for amateur Italian football players (*n* = 1671), except with regard to the risk of abrasion on AT. These results provide some evidence that the type of injury may change the perceived link between playing surfaces and injury and that factors such as ability, country, or surface experience may also influence the perceived links.

The previous questionnaire studies have focussed on NT and AT comparisons which may not adequately represent all countries’ playing surface experience especially those in economic difficulties or challenging climates who may frequently play on alternative surfaces such as gravel or dirt [3]. The questionnaire studies also provided little or no justification for the questions used to assess players’ perception of injury and therefore may not capture all themes regarding an injury. Therefore, elite football players’ perceptions of playing surfaces were assessed in a qualitative study utilising interviews and focus groups to identify important themes which may not be captured in other perception studies [14, 15]. Inductive analysis resulted in a relationship map of the players’ perceptions which defined ‘The Surface’ as the physical entity with associated surface properties and was perceived to influence six aspects of football. ‘Injury and Fatigue’ was one aspect and encompassed four sub-themes of players’ perceptions: ‘Incidence of Injury’, ‘Location of Injury’, ‘Type of Injury’ and ‘Risk Factors’. Key results included a perception by players for them to be at higher risk of getting injured on AT compared to NT, frequent use of terms such as joint soreness which was attributed to the surface hardness or switching between surfaces and feelings of fatigue were linked to soft surfaces [15]. Although the qualitative study and questionnaire studies before offered important insights of players’ experience for comparison with current epidemiological surface injury studies, the small cohorts on which these were based may not represent the overall elite football population and the surfaces they play on. Therefore, it was deemed necessary to explore the central dimensions and sub-themes of injury and fatigue across a wider population to determine whether the attitudes and opinions were shared by players worldwide [15].

The overall purpose of this study was to analyse elite football players’ perceived links between playing surfaces and injury from a worldwide cohort of players. The first objective was to quantify perceptions of the central dimension ‘Injury and Fatigue’ and sub-themes, ‘Incidence of Injury’, ‘Type and Location of Injury’ and ‘Risk Factors’ from a worldwide cohort of players. Using mixed effect binary logistic regression models, a second objective was to identify factors which influence players’ perceived link between playing surfaces and injury. Based on the review of previous literature and the results of the qualitative study, it was hypothesised that:

- H1 A high proportion of players (> 90%) would perceive a link between playing surfaces and injury.
- H2 Perceived links between playing surfaces and incidence and type and location of injury would be closely linked to the surface experience of countries, particularly countries with more NT experience compared to other surfaces.
- H3 Factors such as surface experience, age and surface properties would significantly explain players’ perceived links between playing surfaces and injury.
  The results of this study can help to inform areas for future playing surface research aimed at trying to alleviate user concerns and meet user (i.e. the player) needs.

## Methods

All study activities involving human participants were in accordance with the ethical standards of the institutional ethics committee and with the principles of the 1964 Helsinki Declaration. All players gave their informed consent, and ethical clearance was obtained from Loughborough University Ethical Advisory Committee.

### Questionnaire Study

A questionnaire was developed to capture the world’s elite football players’ perceptions regarding the main dimensions identified in an initial qualitative study [15]. This paper will focus on the questions posed to understand players’ perceptions of the central dimension, ‘Injury and Fatigue’.

#### Participants

According to the FIFA Big Count (2006), there were 112,000 registered professional players worldwide representing six FIFA confederations. Target sample size was determined for each confederation to reflect the distribution of players amongst confederations. A total of 1129 elite players (median age (range) = 24 (18–39 years), 1018 male and 111 female) representing 44 countries completed the questionnaire during the 2012/2013 season. Players were included in the study using a pragmatic non-random cluster sampling approach such that a convenience sample of clubs from all six FIFA confederations could be visited within time and cost constraints [16].

#### Data Collection

Electronic and hard copies of the questionnaire were produced. When possible, a representative from the Sports Technology Institute at Loughborough University was present whilst players completed the questionnaire. The participants were also provided with a supporting document, in the players’ spoken language, to explain the difference between question formats and provide definitions of the surface properties contained in Q4.1.2 and Q4.1.1.2.

**Fig. 1:**
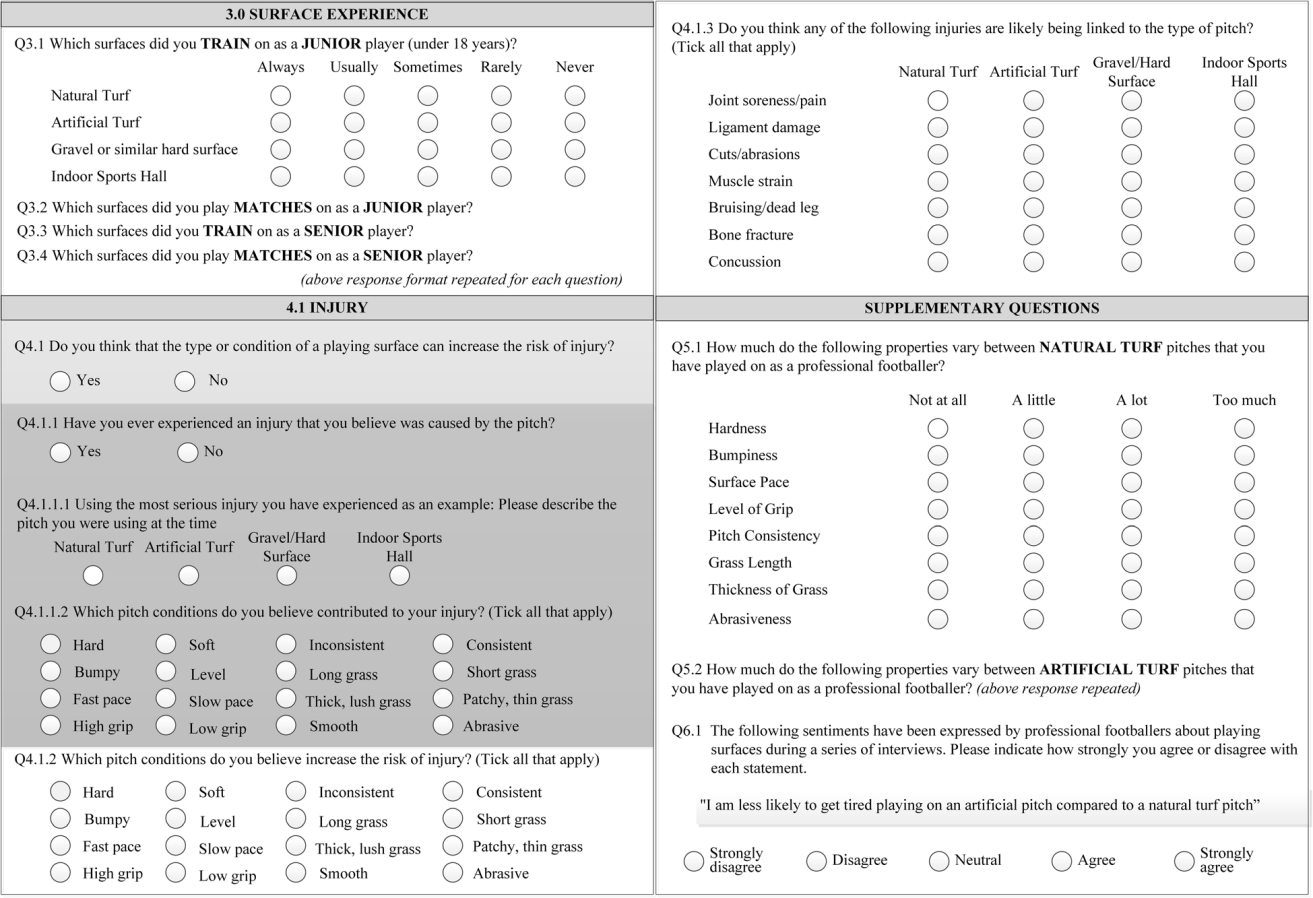
Questions relating to the main dimension ‘Injury and Fatigue’

The questionnaire was divided into sections which covered the central dimensions identified by players during the qualitative study; extracts of relevant sections for this paper are shown in Fig. 1. The first two sections of the questionnaire gathered socio-demographic information. Section three gathered information on players’ experiences of four surfaces (Natural Turf (NT), Artificial Turf (AT), Gravel (GR) and Indoor (IND)) used for training or playing matches as juniors and seniors. Section 4 addressed the central dimension, ‘Injury and Fatigue’ that emerged from the initial qualitative study. Players were asked a starter question (Q4.1); if the player indicated the type or condition of a playing surface was related to injury, then they were asked a series of more detailed questions. Similarly, only if participants responded ‘Yes’ to Q4.1.1 were they asked to respond to Q4.1.1.1 and Q4.1.1.2 (dark grey, Fig. 1). Supplementary questions that appeared in other sections of the questionnaire but were relevant to the data analysis in this paper are also presented in Fig. 1 (Q5.1, Q5.2 and Q6.1).

#### Data Analysis

Questionnaire data was analysed using statistical analysis software R (R Foundation for Statistical Computing, Austria). Initially, players’ perceptions were considered as a whole followed by more in-depth interrogation of the data by surface experience.

**Fig. 2:**
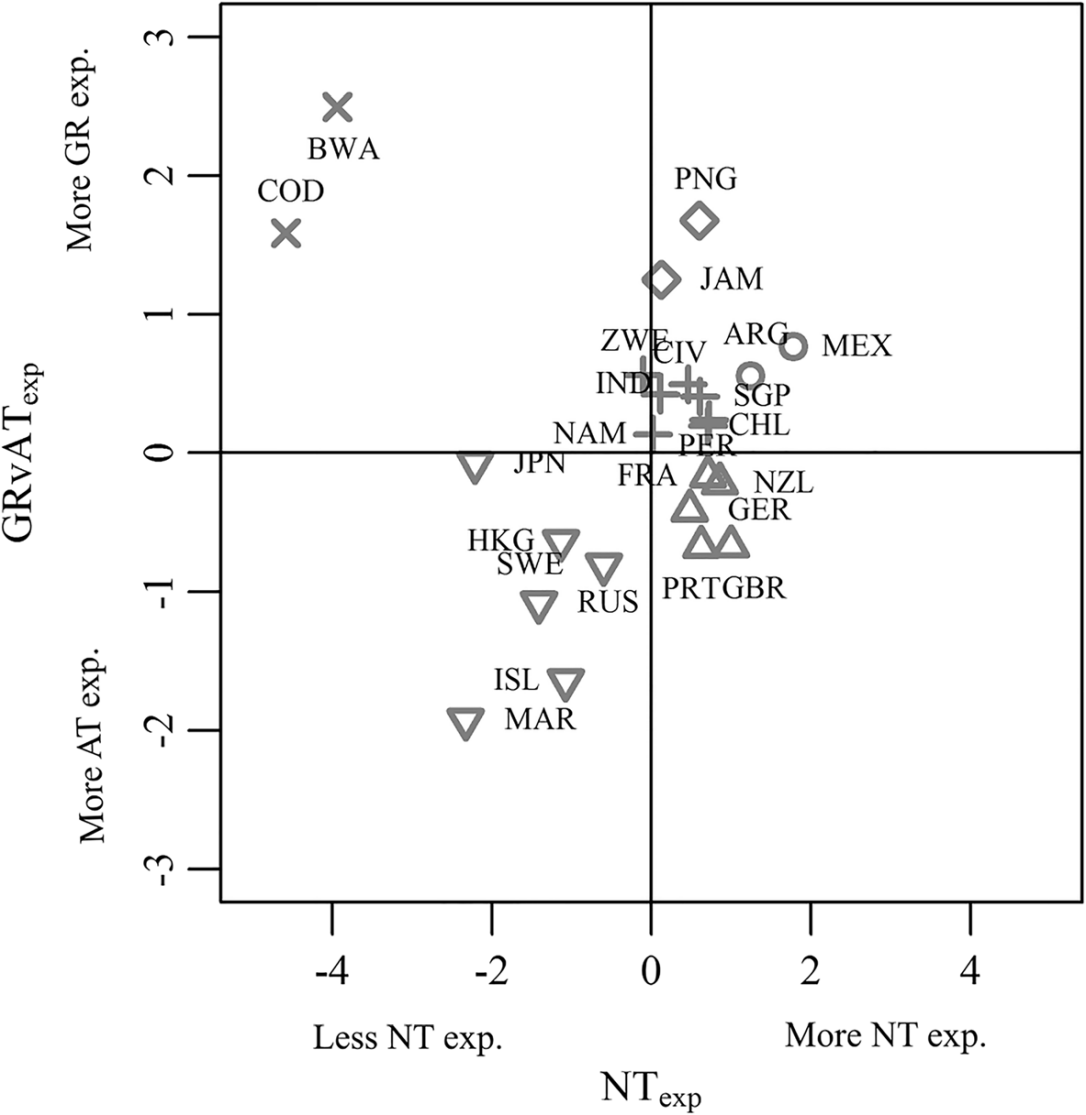
Mean principal component scores representing the surface experience of players from countries with ≥ 20 respondents. Marker types represent the six k-means clusters. Cluster 1 (white diamond) Japan, Hong Kong, Sweden, Iceland, Russia, Morocco; Cluster 2 (white down-pointing triangle) Zimbabwe, Chile, Ivory Coast, Peru, India, Singapore, Namibia; Cluster 3 (multiplication sign) Papua New Guinea, Jamaica; Cluster 4 (white up-pointing triangle) Mexico, Argentina; Cluster 5 (white circle) Botswana, Democratic republic of Congo and Cluster 6 (plus sign) United Kingdom, New Zealand, France, Germany and Portugal

A measure of players’ surface experience was required to provide a more in-depth interrogation of some questions, particularly when exploring factors that explained players’ perceptions. Owen et al. [16] deemed it inappropriate to use the responses of the 16 questions on surface experience (Q3.1 to Q3.4) in mixed effects ordinal logistic regression models due to problems relating to multicollinearity and stability of parameter estimates. To overcome these issues, principal component analysis (PCA), with polychoric correlations, of players’ responses to Section 3.0 questions (Fig. 1) was undertaken to gain a quantitative measure of players’ surface experience. Several principal components (PC1, PC2, PC3, …) and PC scores represented players’ surface experience. Only PC1 and PC2 are considered further (here on referred to as NT_exp_ and GRvAT_exp_) as they largely explained differences in players’ surface experience, whereas the remaining principal components explained differences related to surface experience at various stages in players’ careers (Table 1). These two principal components distinguished between the surface experience of players from different geographical locations as reported in Roberts et al. [17] and shown in Fig. 2. A k-means cluster analysis, based on the mean values for NT_exp_ and GRvAT_exp_, revealed six clusters explained 91.7% of the variance in NT_exp_ and GRvAT_exp_ scores between countries (Fig. 2). Principal component analysis also gained a measure of players’ perceptions of the variability in pitch properties between NT (NT_var_) and AT (AT_var_) surfaces on which they played as a professional (Q5.1 and Q5.2, Fig. 1). For brevity, further details on this analysis are provided in Owen et al. [16].

**Table 1:** Interpretations of the first two principal components and cumulative variance explained [24]

PC	Cum. variance explained (%)	Description
PC1: NT_exp_	46.2	Larger positive values are associated with players who have more experience of NT and less experience of other surfaces such as AT or Gravel, and vice-versa giving larger negative values.
PC2: GRvAT_exp_	66.3	Larger positive values are generally associated with players who have more experience of Gravel and less experience of AT, and vice-versa giving larger negative values.

Associations between categorical variables by surface experience were analysed using *χ*^2^ tests. Correspondence analysis was performed on questions with multiple choice responses such as Q4.1.3 to help find patterns in the contingency table. The two-dimensional correspondence analysis plots were qualitatively inspected to determine the associations between rows and columns based on the proximity of the included attributes.

Finally, three mixed effects binary logistic regression models were used to determine whether the injuries players believe are associated with a particular playing surface (Q4.1.3, dependent variable) could be explained by their perceived risk factors (independent variables). The independent variables included were NT_exp_, GRvAT_exp_, age, playing position, NT_var_, AT_var_ and surface properties (Q4.1.2). Country, included as a random effect, accounted for differences between country clusters and surface experience. A random effect was used firstly, to reduce the number of parameters required to be estimated for the country effects (i.e. from 43 to 1) and secondly because only a sample of professional football-playing countries were included in the study.

## Results

### Injury Overview

In support of Injury and Fatigue being identified as a central dimension in the qualitative study, 91% of the 1129 players who completed the questionnaire believed the type or condition of a playing surface could increase injury risk.

### Incidence of Injuries

**Fig. 3:**
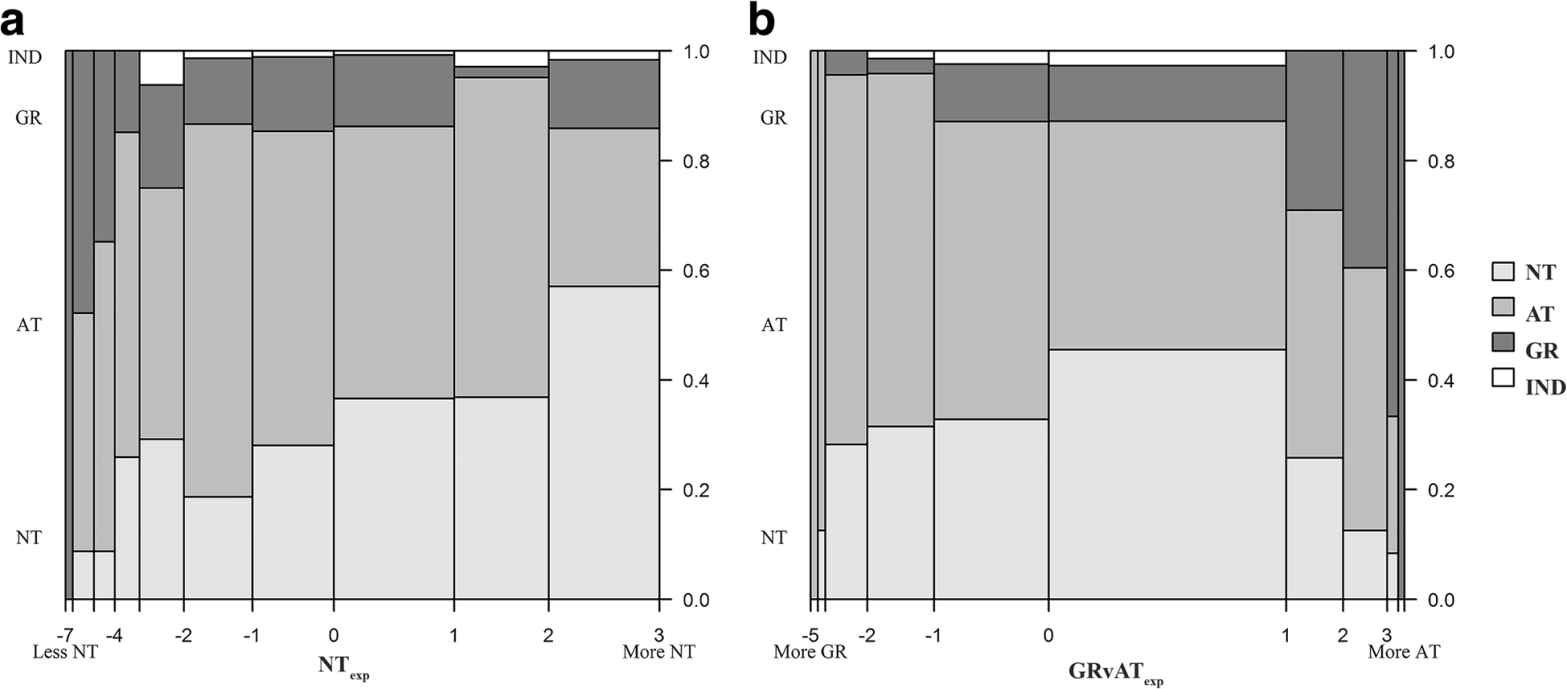
Playing surface where the most serious injury was reported, expressed as a proportion (*Y*-axis) relative to varying levels of **a** NT_exp_ and **b** GRvAT_exp_ (*X*-axis)

Overall, approximately two thirds (64%) of respondents to Q4.1.1 sustained an injury believed to be caused by the type or condition of a playing surface. Of those 650 respondents, the most common surface on which their most serious injury was sustained was reportedly on AT (50% of respondents), followed by NT (34%), then Gravel (14%) and finally an Indoor surface (1%). Interrogating Q4.1.1 by NT_exp_ shows, as expected, players with more NT experience (higher NT_exp_ scores) report NT as the surface on which they experienced their most serious injury (Fig. 3a). A substantial proportion of those with less AT experience (high GRvAT_exp_ scores) still reported a serious injury on AT (Fig. 3b).

**Fig. 4:**
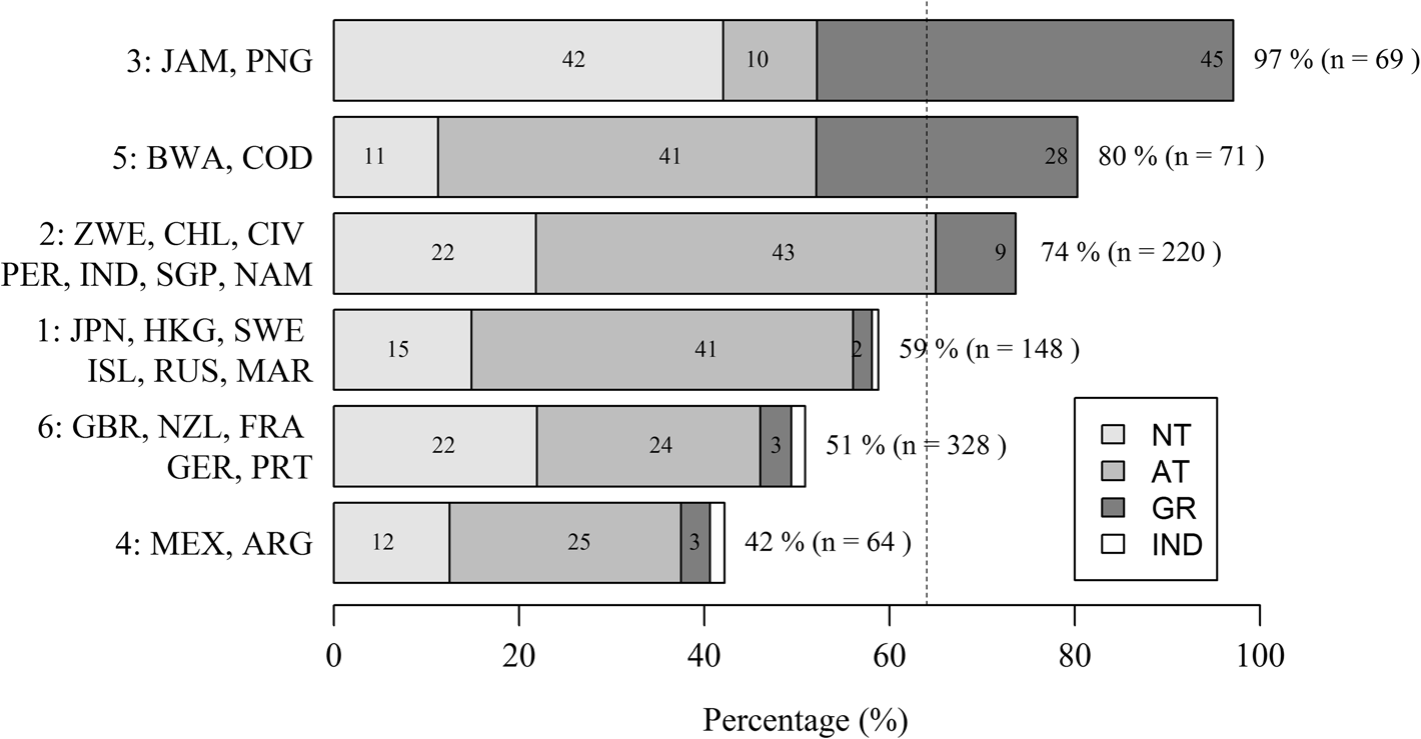
Percentage of players (%) for each country cluster who sustained an injury that they believed was caused by the pitch (Q4.1.1) (order highest to lowest total %) grouped by the type of surface used at the time of injury (Q4.1.1.1) and expressed as a percentage of all responses to Q4.1.1. The dashed line (64%) represents the mean of all players who responded yes to Q4.1.1.

The overall percentage of respondents who sustained an injury believed to be caused by the pitch (Q4.1.1) was significantly related to the country clusters surface experience (*χ*^2^ = 68.04, df = 5, *p* value < 0.05) (Fig. 4). A post hoc pairwise comparison with Bonferroni-Holm correction found a significantly greater percentage of players from cluster 3 (Jamaica and Papua New Guinea) than all other clusters sustained an injury they believed was due to the surface. Figure 4 also shows they were mainly using NT (42%) or Gravel (45%) at the time of the injury. Cluster 5 (Democratic Republic of Congo and Botswana) was also significantly different to all other clusters. Cluster 5 also had a high incidence of such injuries on Gravel (28%) but also a high incidence on AT (41%). There were no statistically significant differences between clusters 1, 4 and 6, thus expressing similar perceptions of sustaining an injury believed to be due to the surface. Cluster 1 countries had the highest levels of AT experience and so this may explain some of the reasons for the higher incidence of injuries on AT.

### Type and Location of Injury

**Fig. 5:**
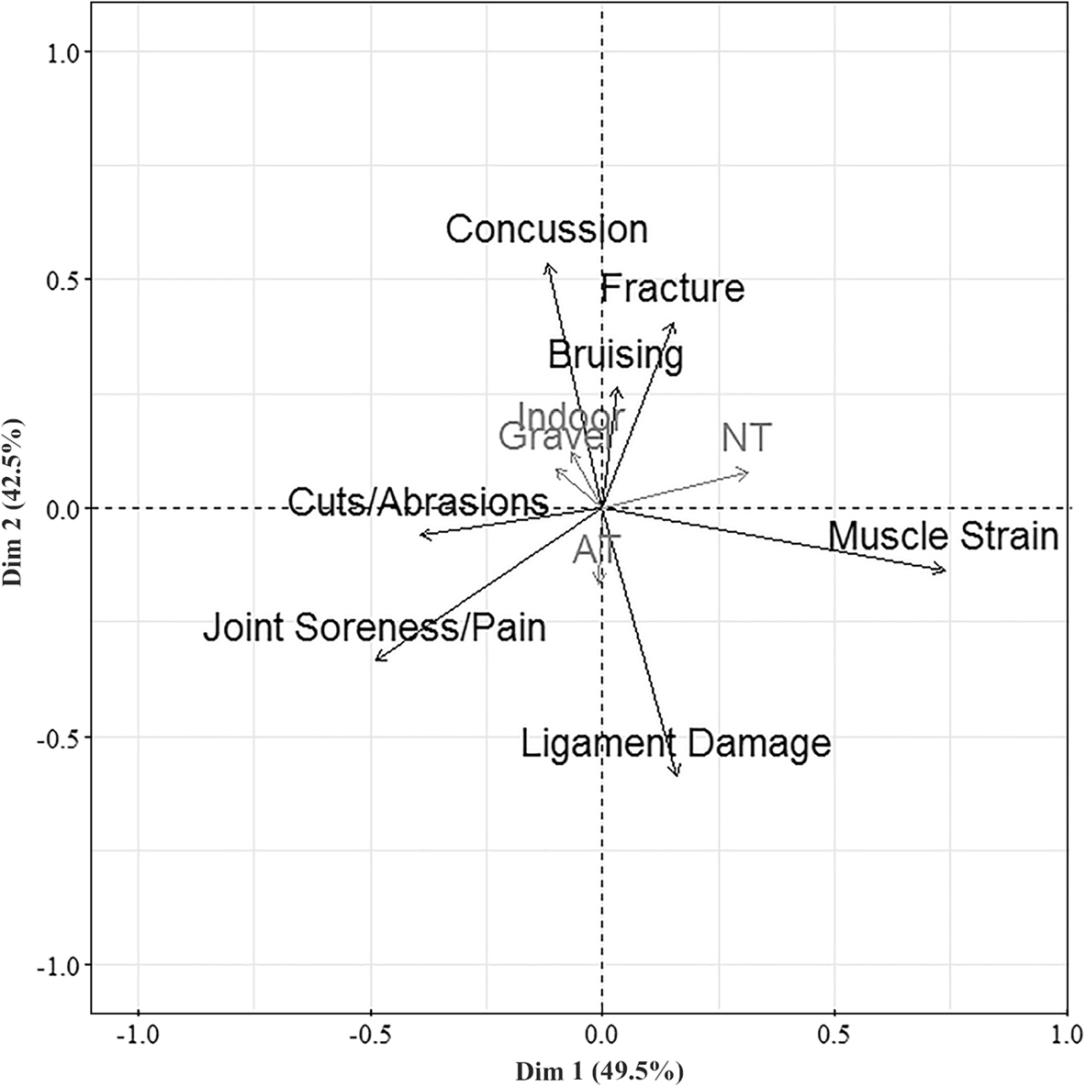
Asymmetric contributing plot of types of injuries (black) linked to different playing surfaces (grey). Playing surfaces that contribute little to the dimensions are close to the centre of the plot (i.e. shorter arrows)

Two dimensions accounted for 92% of the association between the type of injuries and surface type based on correspondence analysis of responses to Q4.1.3 (Table 2, Fig. 5). Dimension 1 explained 49.5% of the information and distinguished between NT and all other surfaces, with larger positive *X*-axis values associated with players linking the injuries more to NT and larger negative values associated with players linking the injuries more to other surfaces. Dimension 2 explained 42.5% of the information and distinguished between AT and all other surfaces, with larger negative values on the *Y*-axis associated with players linking the injuries more to AT and larger positive values associated with players linking injuries more to other surfaces.

**Table 2:** Contingency table of type of injury associated with Natural Turf (NT), Artificial Turf (AT), Gravel or Indoor playing surfaces

Injury	NT	AT	Gravel	Indoor	Total
Joint Soreness/Pain	129	664	485	318	1596
Ligament Damage	209	635	346	212	1402
Cuts/Abrasions	160	558	565	239	1522
Muscle Strain	309	488	310	190	1297
Bruising	202	395	407	247	1251
Fracture	233	374	421	256	1284
Concussion	187	346	421	304	1258
Total	1429	3460	2955	1766	9610

Ligament damage and joint soreness vectors were in the direction of the AT vector and displayed a small angle to the AT vector which suggests AT was mostly associated with these perceived injuries (Fig. 5). Natural turf was mostly associated with Muscle Strain, and the remaining surfaces (Gravel and Indoor) were more associated with Concussion, Fracture and Bruising.

**Fig. 6:**
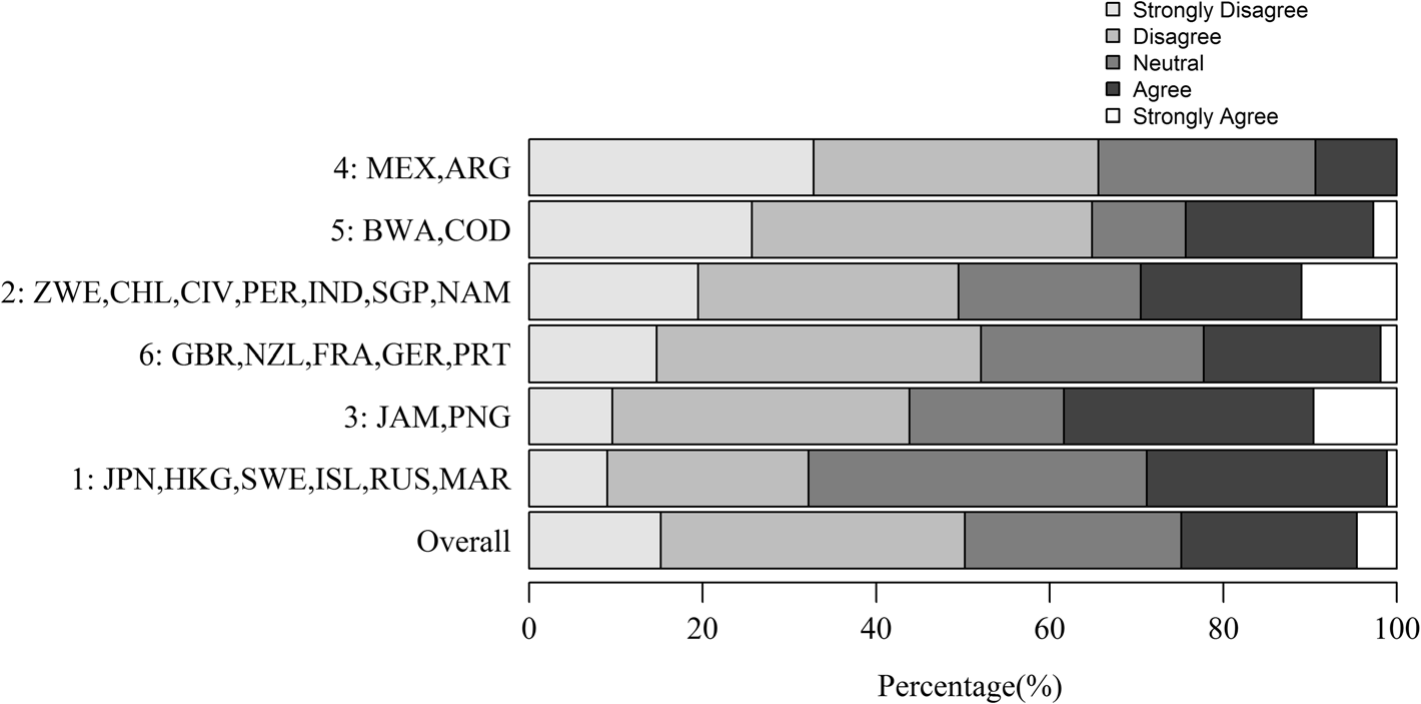
Overall percentage response (%) and response by surface experience cluster (ordered from highest to lowest percentage of ‘Strongly Disagree’) to the statement ‘I am less likely to get tired playing on artificial turf (AT) compared to a natural turf (NT) pitch’

Whilst 50% of players disagreed (strongly or otherwise) they were less likely to get tired on AT compared to NT (Fig. 6), 25% of players were neutral on this issue and 25% agreed (strongly or otherwise).

Cluster 1 (Japan, Hong Kong, Sweden, Iceland, Russian Federation) had the lowest proportion of players disagreeing with the statement in Q6.1 (Fig. 6), and these countries had the greatest experience of AT (Fig. 2). In contrast, cluster 4 (Mexico and Argentina) and cluster 5 (Botswana and Democratic Republic of Congo) had the greatest proportion of players strongly disagreeing or disagreeing with the statement in Q6.1 (Fig. 6), and these countries had the lowest levels of AT experience (Fig. 2).

### Risk Factors

**Fig. 7:**
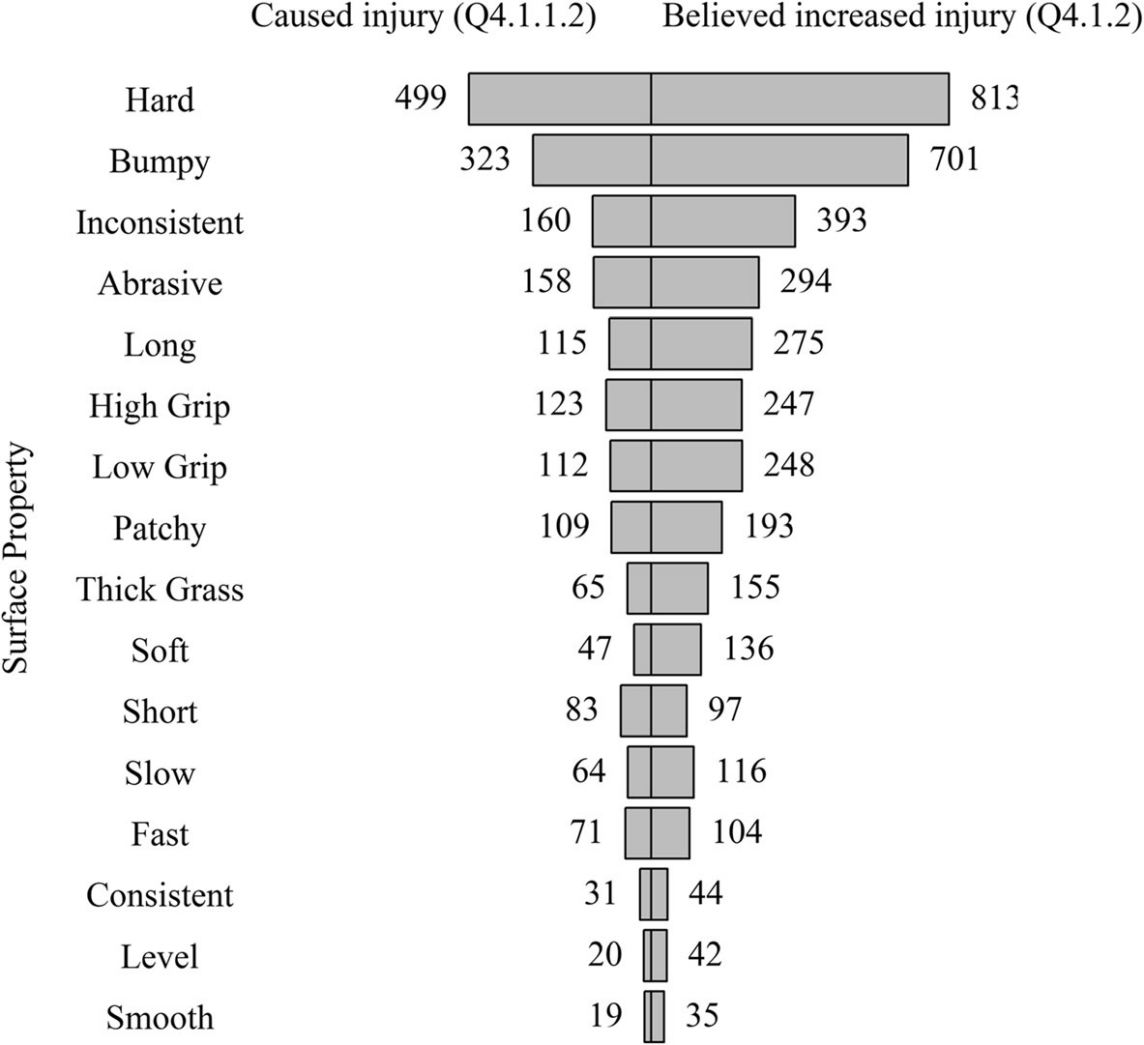
Horizontal pyramid plot of surface properties believed to have contributed to an injury (Q4.1.1.2) and believed to increase the risk of injury (Q4.1.2) ordered by total number of responses for both questions

Similar patterns were observed between the surface properties believed to have caused an injury (Q4.1.1.2) and believed to increase injury risk (Q4.1.2). Hard was frequently selected followed by Bumpy and Inconsistent (Fig. 7).

**Fig. 8:**
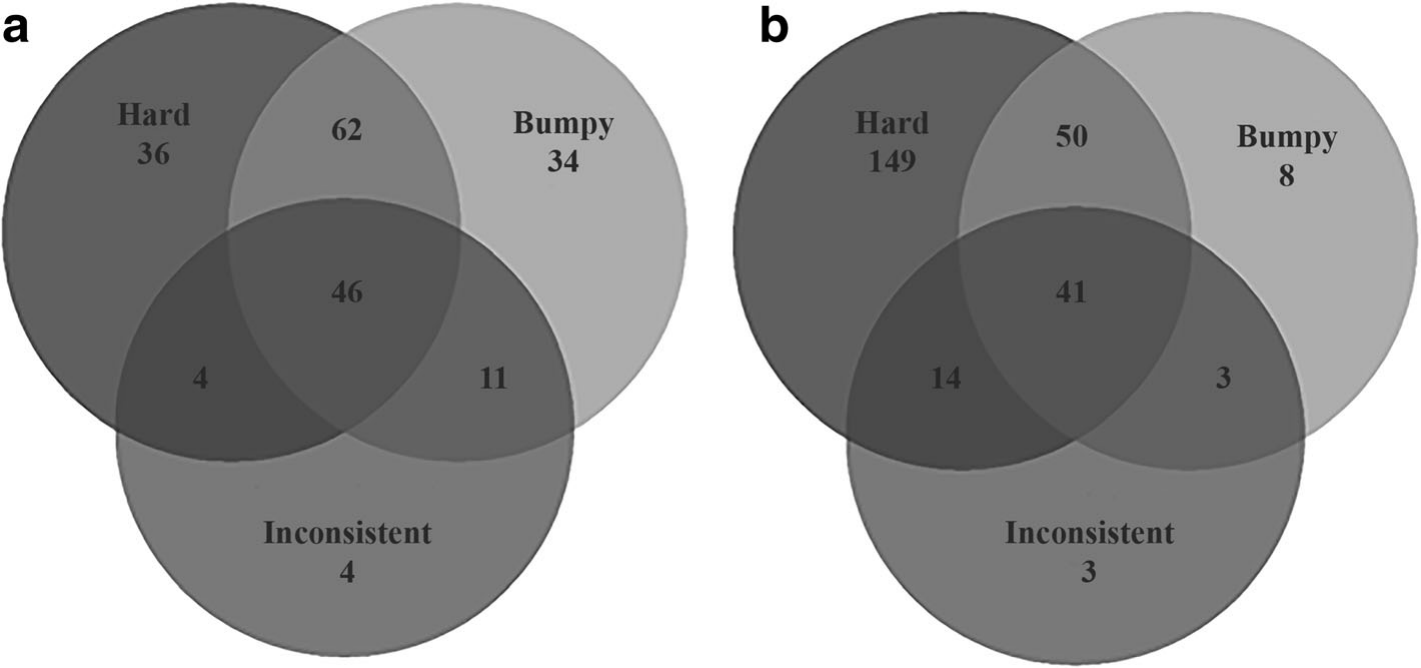
The relationship between the three main surface properties from Q4.1.1.2 that were considered to be responsible for an injury sustained by a player on **a** natural turf (NT) and **b** artificial turf (AT) (Q4.1.1.1)

If the injury was sustained on NT (Q4.1.1.1), players selected hard and bumpy (*n* = 64) or all three properties (Q4.1.1.2) (*n* = 46) (Fig. 8a). In contrast, players who sustained their injury on AT predominantly chose Hard (*n* = 149) suggesting that the hardness of AT surfaces are perceived as contributing to almost all injuries on AT, whereas injuries sustained on NT seem to be perceived as being caused by a wider variation of pitch conditions.

### Modelling Player Perception of Injury and Perceived Risk Factors

With country added to the model as a random effect, players’ surface experience measured using NT_exp_ and GRvAT_exp_ scores were not significant and were excluded. NT_var_ was also not significant and excluded from the model. The final three models explained a statistically significant component of players’ perceptions of the link between injuries and the perceived risk factors (Table 3) with satisfactory values for Nagelkerke’s *R*^2^ (0.24 and 0.29).

**Table 3:** Summary of binary logistic regression models for (i) Joint Soreness/Pain and AT, (ii) Ligament Damage and AT and (iii) Muscle Strain and NT

Outcome	Predictors	*β*	S.E.	*p*	OR	*χ* ^2^	*p*	Nagelkere *R*^2^
Joint Soreness/Pain on AT	Age	0.26	0.08	0.00**	1.32	269.81	< 0.001	0.29
	AT Var.	0.18	0.08	0.02*	1.19			
	Hard	1.11	0.18	0.00**	3.04			
	Bumpy	0.36	0.17	0.03*	1.43			
	High Grip	0.50	0.18	0.02*	1.65			
	Soft	0.82	0.24	0.00**	2.80			
	Inconsistent	0.55	0.18	0.00**	1.73			
	Low Grip	0.58	0.20	0.00**	1.79			
Ligament Damage on AT	AT Var.	0.17	0.07	0.02*	1.19	220.46	< 0.001	0.24
	Hard	1.34	0.17	0.00**	3.84			
	Bumpy	0.69	0.16	0.00**	1.99			
	High Grip	0.81	0.18	0.00**	2.25			
Muscle Strain on NT	Hard	0.86	0.18	0.00**	2.35	229.34	0.01	0.25
	Soft	0.68	0.18	0.00**	1.98			

*OR* odds ratio

**p < 0.01; *p < 0.05

The fitted models suggested a greater tendency for older players to associate Joint Soreness/Pain with AT (odds ratio = 1.32), meaning a player who is 1 year older has an increased odd (1.32 times as high) of associating Joint Soreness/Pain with AT. Table 3 also suggested players who had experienced greater variability in AT surfaces were more likely to associate Joint Soreness/Pain and also Ligament Damage with AT. Players who believed Hardness was a contributing factor to injuries were more likely to believe that Joint Soreness/Pain and Ligament Damage were linked to AT (odds ratios = 3.04 and 3.84 respectively) and also more likely to believe Muscle Strain was linked to NT (odds ratio = 2.35). Interestingly, both Hard (odds ratio = 2.35) and Soft (odds ratio = 1.98) were statistically significant predictors of Muscle Strain on NT.

## Discussion

The overall purpose of this study was to analyse elite football players’ perceived links between playing surfaces and injury from a worldwide cohort of players. The results of this study supported the hypotheses that a high proportion of football players (> 90%) would perceive a link between playing surfaces and injury; perceived links between incidence, type and location of injury varied between countries and factors such as surface experiences, age and surface properties explained players’ perceived links. The results have identified three areas where future research should be focussed to help develop surfaces that alleviate user concerns and meet user (i.e. player) needs: (i) current reporting of soreness, pain or fatigue as injuries; (ii) contribution of surface properties to injury; and (iii) surface experience of players from different countries differentiates their views of injury risk.

### Injury Reporting of Soreness, Pain and Fatigue

The opinion that the type or condition of a playing surface could increase the likelihood of injuries was shared by 91% of the worldwide cohort of football players in this study. This opinion was a similar view shared by the professional North American football players questioned in Poulos et al.’s [2] study. A limitation of Poulos et al.’s [2] study was the small sample size; however, this study has discovered several common themes based on a wider population. Soreness, aches and pains were frequently mentioned by players in the qualitative study and described as problems lasting only a few days [18]. In this study, joint soreness and pain were associated with artificial turf (Fig. 5). Soreness has been highlighted as a problem for players in previous injury perception studies [2], yet reporting soreness as an injury in official medical reports may not always be achieved due to the definition of injury. Timpka et al. [19] commented on the limitations of current sports injury reporting systems due to their conceptual basis and instead use the term ‘Sport Impairments’ and provide definitions of injuries that represent varied perceptions of health services, athletes and sports institutions [19]. The present study supports this concept as the physical problems players identified may not be adequately captured in current reporting systems of football injuries that focus on injury from a clinical perspective. The physical complaint may prevent players from training at a high intensity in subsequent sessions and could be a player’s indication of the substantial muscle function loss and central nervous system impairments which have been found up to 48 h after a competitive football match on AT [20].

### Contribution of Surface Properties to Injury

Hard, bumpy and inconsistent were surface properties perceived to have contributed to an injury on both NT and AT, rendering surface properties equally as important to consider as surface type when reporting injuries. Players strongly linked ligament damage to AT (Fig. 4 and Table 3) which contrasts some studies reporting no increase in the likelihood of sustaining a serious injury between surface types [7]. Exploring players’ perceptions of ligament damage on AT and muscle strain on NT, the regression model interestingly showed both ‘Hard’ and ‘Soft’ surface properties to be predictors for these perceived injuries suggesting neither surface property was suitable. A similar outcome was found for high and low grip. Surface properties are identified as potential risk factors for injury in several sports [2, 21]; however, surface conditions are often based on subjective assessment [12]. Given the link between injuries and surface properties, regardless of surface type, there might be a need for NT surfaces to be tested and meet the requirements of an industry standard similar to the FIFA Quality Programme for Football Turf, which to the authors’ knowledge does not currently exist. The methods used to assess the hardness or grip of a playing surface will be important to consider in the future given that subjective ratings of subtle surface hardness differences show low agreement to objective measures of ground hardness [11, 12]. Hence, to reduce injury risk, the surface properties should be considered from objective (i.e. engineering) and subjective (i.e. player) perspectives [22]. Objective and subjective surface property differences could then form the focus of injury comparison studies rather than between discrete surface types.

### Differences Between Countries with Varying Surface Experience

The percentage of players experiencing an injury believed to be due to the type or properties of a surface were greater for countries with more experience playing on Gravel (i.e. higher GrvAT_exp_ scores) compared to more NT experience (i.e. higher NT_exp_ scores). The differences in surface experience between countries could be due to a plethora of underlying factors, such as wealth of a country and quality of available pitches or climate, as examples [2], but players’ experiences clearly influence their perception of injury. Surface experience differences between countries should be acknowledged when interpreting previous studies that have used a subset of players from specific countries and should be considered as an important variable to measure in future injury studies. Future research studies could target the specific results for perception of injury and playing surface based on each cluster of countries. For example, countries where players indicated the sustained injury was on Gravel suggest they were more exposed to poorer quality surfaces and future studies reporting incidence of injuries should have adequate measures of surface quality based on several surface property performances. Alternatively, countries with little AT experience and predominantly NT experience indicated strong opinions when directly commenting on feelings of fatigue on AT (Fig. 5). Follow-up studies to discover how these opinions were formed despite minimal experience could help alleviate unsubstantiated opinions of players in the future.

### Limitations

This study relied on players’ perceptions of injury and was not backed up by official medical records. Therefore, prejudiced opinions or errors in recalled memory may be captured. The length of time over which participants were asked to recall injuries was not controlled in the questionnaire. The questions regarding players’ recall of injuries were, overall, not overly detailed, instead requesting simple information about whether they had been injured (Yes or No) and the surface they were using at the time of their most severe injury from four options. When questions about injury recall become more detailed, such as the number of injuries or location of injury, a reduction in overall recall accuracy compared to prospective injury surveillance has been reported [23]. We believe that the simple questions included in this questionnaire would have been less susceptible to recall bias. The one question that could be susceptible to increased recall bias due to the increased level of detail was Q4.1.1.2. However, there was close agreement between Q4.1.1.2 and Q4.1.2 providing some confidence that whether there were errors in recall or not, the surface properties which caused injury were still perceived to be linked to injury. Furthermore, even possible prejudiced opinions can reveal interesting insights and areas for future research. Although a worldwide cohort of players was studied, it still does not account for every professional footballing nation but provides the most comprehensive to date.

## Conclusions

A worldwide cohort of elite association football players largely believed the type or condition of a playing surface could increase injury risk. Players were concerned with the soreness, aches and pains experienced on different playing surfaces which may not be adequately captured in literature or injury reports. Injuries such as ligament damage were closely linked to AT despite current literature not supporting this perception. Surface properties partially explained the perception of injuries linked to a particular surface, yet future studies should better capture surface properties objectively and subjectively to form the focus of injury comparison studies rather than discrete surface types. Surface experience measures differentiated between footballing nations’ perceptions of injury and could help direct future research studies.

## Abbreviations

AT: Artificial turf
NT: Natural turf
